# Reference and Solution Architecture for GenAI- and GIS-Enhanced Physical Activity Interventions: Towards Implementing the AI4Motion Platform

**DOI:** 10.1007/s10916-025-02269-x

**Published:** 2025-10-30

**Authors:** Michal Doležel, Radim Lískovec

**Affiliations:** 1 Consumer Health Informatics Lab, Department of Information Technology, Prague University of Economics and Business, Prague, Czech Republic; 2 Department of Human Movement Studies, University of Ostrava, Ostrava, Czech Republic; 3 Department of Geography, Masaryk University, Brno, Czech Republic

**Keywords:** LLM, LBS, API, LLaMA, Digital Health Intervention, Geofencing

## Abstract

Digital Behaviour Change Interventions (DBCIs) aim at improving individual health by engaging various means of Information and Communication Technology (ICT), including mobile apps and wearables. Participant intervention fatigue may happen when DBCIs become too frequent, repetitive, demanding, or lack perceived relevance, and this may result in participants’ reduced motivation and adherence over time. Advancing technology-supported engagement mechanisms is therefore of utmost importance. To address this problem, we present a reference and solution architecture based on open-source technologies and open Application Programming Interfaces (Open APIs). First, we integrated a Large Language Model (LLM) component into the DBCI design. Second, to support context-awareness, we enhanced this integration by adding a Geographic Information Systems (GIS) element. Our pilot implemented AI4Motion platform targets both personalization and contextualization aspects of DBCIs. Our work contributes to the emerging discussion on LLM/GIS-related system design patterns for digital platforms supporting Ecological Momentary Assessment (EMA), Experience Sampling Method (ESM), and Just-in-Time Adaptive Interventions (JITAIs).

**Supplementary Information:**

The online version contains supplementary material available at 10.1007/s10916-025-02269-x.

## Background

Digital Behaviour Change Interventions (DBCIs), a subfield of Digital Health Interventions (DHIs), apply information and communication technologies (ICT) to support health behaviour change [1]. While DBCIs are primarily grounded in health, behavioural, and human movement sciences, such as behavioural medicine, physical activity psychology, and kinesiology [2, 3], they increasingly benefit from human-centred design efforts and digital health systems research [1, 4]. Notable past efforts focused on integrating mobile apps, wearables, and smart sensors [2]. Methodologically, two key approaches in physical activity research that heavily rely on these technologies are ecological momentary assessments (EMAs) or experience sampling methods (ESMs), and just-in-time adaptive interventions (JITAIs) [3].

An overall aim of DBCIs using these methods is to support habit formation, often through behavioural nudges such as short text messages or push notifications [5]. However, overly frequent, repetitive, or irrelevant nudges can lead to intervention fatigue, reducing participant engagement and adherence. Designing for personalization and sustained motivation, and gradually building the “science of engagement” [2], is therefore critical [1, 5]. Among emerging technologies, generative AI (GenAI) shows promise for enhancing engagement and relevance in DBCIs [2], and, eventually, for bringing a significant shift in the field of behavioural medicine [6]. In addition, some researchers in this field have suggested employing health geography and geographical information science, including location-aware and geofencing technologies, for DBCI tailoring [7, 8]. In our paper, we propose how to employ GenAI and location-aware technologies in concert.

First, Large Language Models (LLMs), a form of GenAI, employ natural language processing and machine learning to support a range of health- and behaviour- related outcomes [2] [6]. In the context of health behaviour change, behavioural healthcare and social science research have begun exploring LLM capabilities. Among other LLM application research streams, such as patient education and symptoms assessments [2], the one covering LLM-based chatbots [9] and JITAI content generation [10] is notable. This includes the use of advanced prompting strategies based on chains of models [9] and comparisons of different models’ generated content [11]. Such studies are often grounded in behavioural science frameworks such as COM-B–which models Behaviour (B) through Capability (C), Opportunity (O), and Motivation (M) [11]–or Motivational Interviewing [9]. Crucially, within JITAI research, a paradigm shift has been proposed: to replace fixed, rule-based logic for message generation with the more flexible, adaptive logic enabled by LLMs [12].

Second, Geographic Information Systems (GIS), as mapping and spatial analytics technologies, enable context-aware and place-sensitive personalization in DBCIs [7]. A key process in LLM-supported DBCIs is the automatic generation of place descriptions from geographic coordinates [13], enabling semantic location labels (e.g., home, supermarket) and integration with location-based services (LBS) (e.g., points of interest, weather, air quality). Furthermore, participant location (e.g., via geofencing) enables better timing of messages or nudges (e.g., in relapse-prone environments), while awareness of participant routines and environmental conditions further tailors DBCIs (e.g., suggesting indoor activity during bad weather) [8].

However, while both behaviour-oriented and system design studies [4] involve complex prompting strategies, they adopt quite a simplistic system design logic. Typically, they rely on ChatGPT or a similar commercial technology to generate the JITAI content, i.e., messages or nudges, via standard web interfaces. Integration mechanisms such as open Application Programming Interfaces (APIs), an important technological landmark in behavioural health [2], appear to be underused. Another key motivation for platform-oriented LLM research is the need to explore the potential of open-source LLM models, such as LLaMA and its health-focused derivatives [14]. Open-source models are likely to be central to digital health systems due to their compatibility with validated environments and their improved controllability and safety, both essential for adoption [2]. Similarly, literature on LLM-GIS integration mechanisms in the DBCI context is scarce.

Bridging behavioural research and digital health systems research, this brief report introduces a minimalistic reference architecture and a solution architecture with LLM and GIS capabilities. Our architecture was preliminary implemented within a private cloud platform. Our overall aim was to employ these innovative technologies for personalized feedback generation, so that effective DBCI engagement and adherence can be ultimately increased.

## Reference and Solution Architecture

**Fig. 1:**
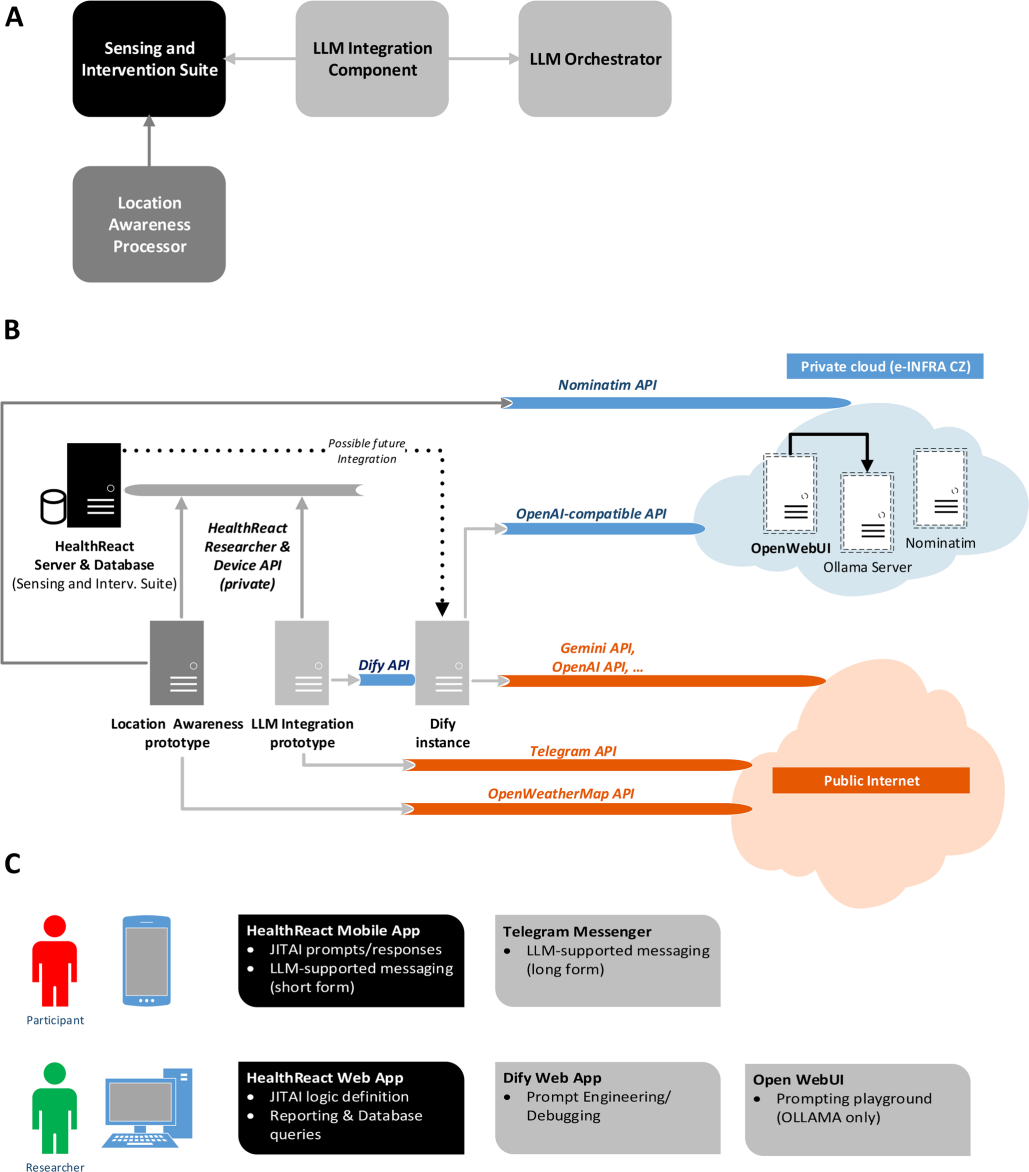
Architecture of the AI4Motion Platform. (**A**) Reference Architecture presents four key components: Sensing and Intervention Suite, LLM Integration Component, LLM Orchestrator, and Location Awareness Processor. (**B**) Solution Architecture shows HealthReact as the Sensing and Intervention Suite, managing wearable and mobile sensor data via two open APIs. EMA logic is triggered using event-based rules and micro-randomised conditions. The LLM Integration prototype (our contribution) retrieves physical activity data, while the Location Awareness prototype (our contribution) generates contextual data. Dify (LangGenius, Inc.) serves as the orchestrator and routes prompts to either external APIs (e.g., OpenAI, Gemini) or local models. Ollama (open source), deployed in a private cloud, hosts these local models. As Ollama’s native API lacks token-based authentication, OpenWebUI (Open WebUI) is used to expose a secured RESTful interface. The Location Awareness prototype uses Nominatim (open-source) and OpenWeatherMap (OpenWeather, Ltd.) APIs to enrich location data with semantic and environmental labels (e.g., place type, weather). These are stored back into HealthReact for use in message generation or researcher feedback. (**C**) Application Tier includes the mobile app for delivering short messages and Telegram for long-form GenAI-enhanced content. Researchers interact via a web interface. Dify and OpenWebUI support prompt testing and design. Italicised terms in the figure denote open APIs and system components. The overall design follows a modular, service-oriented approach

*Reference architecture* serves as a conceptual, generalizable framework. Our minimalistic proposal (Fig. 1a) consists of four principal components: (i) *Sensing and Intervention Suite*, (ii) *LLM Integration Component*, (iii) *LLM Orchestrator*, (iv) *Location Awareness Processor*. *Sensing and Intervention Suite* implements the main EMA/ESM/JITAI logic, including nudges generation and experience samples collection. The suite is expected to have both a server part (application server, database system, researcher web interface) and a client part (participant mobile app). *LLM Integration Component* implements a translation capability, i.e. it queries the *Sensing and Intervention Suite* to obtain participant data such as physical activity indicators and location-based data, pre-process them, and subsequently generates a LLM prompt sent to *LLM Orchestrator* via an open API. Finally, the *Location Awareness Processor* generates semantically meaningful textual descriptions of the environment based on a participant’s raw location measurements, such as labelling a place as ‘Home’ or ‘Work’, or providing contextual information like the weather forecast for the current city/area or nearby landmarks. These are, via an open API, then stored in *Sensing and Intervention Suite* and used as short-text or long-text nudges, or as internal summaries for researchers.

In contrast to reference architecture, *solution architecture* of the AI4Motion platform is a concrete realization of the proposal above, which was extended to the context of our DBCI research project and later pilot-instantiated into a private cloud implementation. The solution architecture displayed in Fig. 1b thus reflects several design constraints, such as the necessity of integrating with the existing EMA/ESM/JITAI software platform chosen by the behavioural researchers earlier. For an overview of the design constraints and brief description of the DBCI project see on-line Appendix 1. Our solution architecture components are visualized in Fig. 1b and detailed in the figure title, where also additional implementations details are provided where appropriate. Given its reliance on open APIs and relatively loose integration, this type of architecture can also be labelled as component-based, service-oriented architecture.

When designing the solution architecture, we considered two variants of the use-case, as follows:

The target platform should provide personalized, engaging, and geocontext specific feedback about the participant’s physical activity on a daily/weekly basis, in two forms:

1. a short-text form, using an EMA/JITAI participant mobile app;
2. a more elaborate form, using a widely used mobile messaging app.a more elaborate form, using a widely used mobile messaging app.

Given the local context, we used *HealthReact* (https://www.healthreact.eu/) in the role of *Sensing and Intervention Suite*. *HealthReact* is a software suite that includes both a server-side system and a participant mobile app available for iOS and Android devices. A more comprehensive and DBCI-contextualized description of HealthReact context is provided elsewhere [15]. As for the *LLM Orchestrator* and *Location Awareness Processor components*, we designed their logic in an iterative manner by own effort, resulting in a Python and R research prototype, respectively. Regarding the fourth component, LLM Orchestrator, we chose *Dify* (LangGenius, Inc.) due to its flexibility for operating with different LLM platforms and providers. This considerably simplified the complexity of the scope of *LLM Integration Component* prototype development.

## Pilot Implementation

**Fig. 2:**
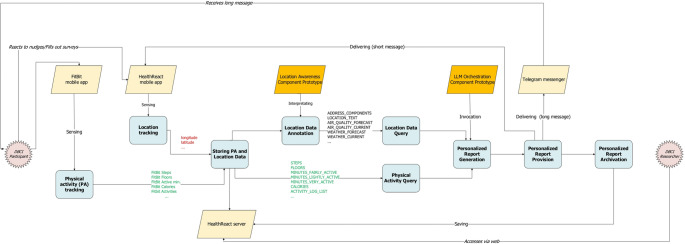
Data Flow Diagram for the LLM and GIS enhanced features. Actors are represented using starburst shapes, and their interactions with the system are italicized. Processes are visualized in blue round-shaped boxes, and system components in parallelograms. The two new components developed as prototypes are marked in orange, while the others in light yellow. The variable naming convention is based on the current HealthReact database structure. From left to right: The Fitbit mobile app is paired with a wearable (e.g., Fitbit Charge 6) used by the DBCI participant, senses physical activity (PA) data, and stores it in the Fitbit cloud. The HealthReact app is installed on the smartphone, sensing raw location measurement data (longitude and latitude). The subsequent process, Storing PA and Location Data, stores these data in the HealthReact database. Next, the two newly introduced component prototypes are involved. The process of Location Data Annotation is carried out by the Location Awareness Component prototype. The prototype retrieves participant coordinates and writes semantic location information, infusing the location data back into HealthReact (not visualized). Subsequently, the LLM Orchestration Component prototype employs this data (Location Data Query) and makes a further query for the PA data (Physical Activity Query). By invoking Dify and a specific LLM, such as Gemma (not visualized), the component generates a personalized report (Personalized Report Generation), which is then provided to the participant (Personalized Report Provision) as a personalized message via the HealthReact mobile app (concise variant) or Telegram Messenger (long variant), and archived back to the HealthReact server

Pilot implementation was carried out using a combination of virtual machines infrastructure (HealthReact) and a private cloud environment e-INFRA CZ (LLM components). We present the data flow diagram in Fig. 2, where the details concerning the data processing are provided. Finally, screenshots of the researcher’s HealthReact interface and the participant’s mobile app, displaying two LLM-generated personalized report, are available in on-line Appendix 1.

## Discussion

The presented work is interdisciplinary in nature. First, our results illustrated a possible strategy for how LLM and GIS elements can enrich DBCIs. Specifically, we demonstrated that LLM-enriched DBCIs do not need to rely solely on commercial LLM technologies such as ChatGPT, given the increased needs for transparency, explainability, and safety [2]. Second, this work highlighted the potential of open APIs for DBCIs. Specifically, we presented one of the possible architectural patterns. Finally, we proposed how GIS can be operationalized beyond traditional spatial analysis in geographical information science; namely, as a contextual awareness layer for DBCI in real-world settings [7].

This brief report clearly has its limitations. First, we were able to highlight only the key technological features and could not provide more detail. In that sense, we acknowledge that our reference architecture is very minimalistic and may not align with all existing EMA/JITAI “no-code” platforms available in the market [3]. Also, the description of pilot implementation provided only limited insights. Second, although the solution architecture was instantiated and pilot implemented as the AI4Motion platform beta, the on-line appendices contain only initial evidence such as the prompting strategy and examples of generated message. That means, we did not present any strong evaluation data, neither of a technical nature nor in terms of human-centred assessment by DBCI participants or health behaviour experts.

We plan to deploy the AI4Motion platform in an AI-enhanced pilot DBCI study, following ethical clearance and participant recruitment. In doing so, we aim to demonstrate more thoroughly how the integration of GIS-informed spatial reasoning with LLM-driven personalization can advance context-aware feedback in DHCI beyond current implementations.

## Supplementary Information

Below is the link to the electronic supplementary material.

Supplementary Material 1(PDF 544 KB)

Supplementary Material 2(PDF 80.4 KB)

## Data Availability

No datasets were generated or analysed during the current study.
