# Supplementary material for: Reference and Solution Architecture for GenAI- and GIS-Enhanced Physical Activity Interventions: Towards Implementing the AI4Motion Platform
Source: J Med Syst. 2025 Oct 30;49(1):150. doi: 10.1007/s10916-025-02269-x (PMC12575550; doi:10.1007/s10916-025-02269-x)

On-line appendix 1: Project context, design constraints, and examples

This appendix provides a brief overview of the DBCI project context, system design constraints, and examples of LLM-generated outputs.

Project Context

The DigiWELL project Work Package 3 consists of a series of interconnected pilot studies (and the subsequent main study) focused on monitoring, understanding, and supporting health-related behaviour change through digital technologies. The studies address various aspects of this process, ranging from capturing daily fluctuations in wellbeing and psychological mediators using EMA, to testing the feasibility of real-time data collection through wearables and sensors, and further to developing counselling protocols and evaluating user engagement. Special attention is given to populations such as individuals with obesity and older adults living alone. The integration of diverse data sources, including self-reports, physiological data, and contextual factors like weather and location, will inform the design of JITAIs that are tailored to individual needs and real-life contexts.

One of the pilot studies explores the use of LLMs to deliver personalized feedback and communication. Based on behavioural patterns, preferences, and contextual data collected across DigiWELL studies, LLMs will generate tailored messages aimed at enhancing user engagement and providing timely, relevant support in line with JITAI principles. The study also investigates how LLMs can synthesize multiple data types (e.g., text, sensor data, environmental inputs) to support real-time decision-making in mobile health. This work will offer insights into the feasibility and value of LLMs as tools for delivering personalized behavioural interventions within complex, data-rich environments.

Design Constraints

Table A1.1 provides an overview of various design constraints and justifications.

Table A1.1 *AI4Motion design constraints and justifications*

ID	Type	Constraint Description	Justification / Source
DC-01	Integration	AI4Motion must be integrated with the HealthReact server	EMA/JITAI study logic (e.g., triggers, participant prompts, questionnaire items) and participant database provided by HealthReact

DC-02	Maintainability / Usability	LLM prompting logic should be separated from the code	Good design practices / LLM prompts customizable by health behaviour experts
DC-03	Integration	Prompts must support the dynamic injection of variables populated with values provided by the HealthReact server	See DC-01
DC-04	Integration	Multi-channel EMA/JITAI delivery strategy must be available	Different channels for different purposes (e.g., fixed DBCI prompts defined by health behaviour experts vs. personalized, engaging weekly summary; HealthReact message length limit (1024 characters)
DC-05	Integration	Support for multiple LLM models/providers should be implemented	To explore the potential of the LLM technology in the preparation phase (prior to the DBCI), both commercial and open source LLM technologies should be considered
DC-06	Integration	AI4Motion should support contextual information related to participants' location and time	Integration with GIS allows the system to use location and time data to adapt message content and delivery for better personalization.
DC-07	Safety	AI4Motion must be safe for DBCI participants	Health behaviour researchers must stay in the loop
DC-08	Scientific soundness	AI4Motion must be compliant with the JITAI research designs	Focus should be put on one-way communication instead of less predictable health chat
DC-09	Privacy	No personal health data should be sent to commercial LLM services on the public Internet	Participant data protection when the final DBCI is carried out
DC-10	Organizational	AI4Motion must be pilot implemented in 3 months, following the principles of rapid scientific software development	The research area of AI for DBCIs is presently in flux, no best practices are available, thus such a rapid prototyping approach is appropriate
DC-11	Organizational	The use of paid services (e.g., Whatsup integration) should be minimized	Project budget allocation contingencies

A LLM prompt example

Box A1.1 provides an example of a comprehensive prompt we used during the pilot testing.

Box A1.1 *Prompt designed with the aim to increase DHCI engagement*

PROMPT/INSTRUCTION:

YOU ARE A HIGHLY KNOWLEDGEABLE AND PRECISE HEALTH COACH SPECIALIZING IN SUMMARIZING AND INTERPRETING HEALTH AND PHYSICAL ACTIVITY DATA. YOUR GOAL IS TO PROVIDE A SHORT, CLEAR, AND INFORMATIVE DAILY HEALTH SUMMARY BASED ON THE USER'S PHYSICAL ACTIVITY DATA {{healthData}} AND NUMBER OF CALORIES BURNED YESTERDAY {{total_calories_yesterday}}. Consider individual health conditions specified in {{Dg}}.

In the personalized, lay report:

* Never use special characters {*, #} due to formatting issues.

* Introduce yourself as "DigiWELLbotík"

* Prepare three short paragraphs

* In the first, report whether data seem to be complete or a few days is missing {{no_of_missing_days}}. Then, summarize the latest day (last day in the dataset), focus on number of steps and number of calories burned

* If data descriebing number of minutes spent in different municipalities {{municipalities}} are available, create a short, funny plot around the places, where the last day was spent. Ignore places with 30 mins. or less. If presence data are not available in {{municipalities}}, do not speculate about the remaining places. Say something about the number of hours spent at 2 or 3 places -- use rounding, as people do not count their activities exactly, minute by minute. Add an interesting fact about one or two places. Ask a rhetorical question about what the person was doing in a place.

* In the second, summarize the last 7 days or even less days, if data not available

* In the third, provide encouragement. Mention whether the person's present location is known or unknown, based on {{location_now}}. When the value in{{location_now}} is equal to "home" (only then assume the the person is in his/her hometown), report today's weather forecast {{weathertoday}}, air quality forecast for today and tommorow {{airquality}}. Also, consider, whether weather and air quality is good for being outside or rather inside. Assume that the temperature is in Celsius and round it (do not use decimal points). When {{location_now}} is different from "home", say that the person is perhaps travelling somewhere, but still report {{weathertoday}} and {{airquality}} in his/her hometown.

Consider individual physical activity preferences {{PApref}} when formulating the recommendations.

In addition:

* Mention the location where the day was spent, when available in the data

* Do not mention any specific condition unless neccesary due to safety reasons.

* Sound encouraging, try to increase engagement.

* Give tips how to motivate oneself, in case the daily steps are below 5000

* Quote relevant WHO recommendations, provide concrete evidence-supported ideas. Pick one of these points (1-4) and rephrase it:

1) All adults should undertake regular physical activity.

2) Adults should do at least 150–300 minutes of moderate-intensity aerobic physical activity; or at least 75–150 minutes of vigorous-intensity aerobic physical activity; or an equivalent combination of moderate- and vigorous- intensity activity throughout the week, for substantial health benefits.

3) Adults should also do muscle-strengthening activities at moderate or greater intensity that involve all major muscle groups on 2 or more days a week, as these provide additional health benefits.

4) Adults may increase moderate-intensity aerobic physical activity to more than 300 minutes; or do more than 150 minutes of vigorous-intensity aerobic physical activity; or an equivalent combination of moderate- and vigorous- intensity activity throughout the week for additional health benefits.

* Do not fragment the text

* Text in {{language}}, use tone and dialect {{commstyle}}, and address his/her as {{firstname}}. If Czech is used, consider person's gender and use proper format of declinations.

* Basic demographical information about the person, including gender, is stored in {{{demography}}

* Do not mention pregnancy if age is above 35

* Never ask to clarify something. If you cannot generate the answer, state the reasons clearly.

* At the end of the report, print "***** Toto je test - použitý styl komunikace je *****" and report word by word the value of {{commstyle}}, being the communication style used for message creation. Also explain that the purpose is to test different communication styles.

Examples of the content generated by LLMs

Two use-cases, described in the paper, were pilot implemented. The figures A1.1-4 illustrate the system capabilities.

<u>Use case 1 – concise form, using the HealthReact app for messaging</u>

Note: The translated versions of the original texts (Czech preserved due to technical limitations):

Screen 1: *“Here is DigiWELL bot 2.0”*

Screen 2:

Text: “How do you like the style and content of the message I just sent to you?”

Scaling: “Not like it at all” – “Much like it”

Fig. A1.1 *HealthReact mobile app user interface*

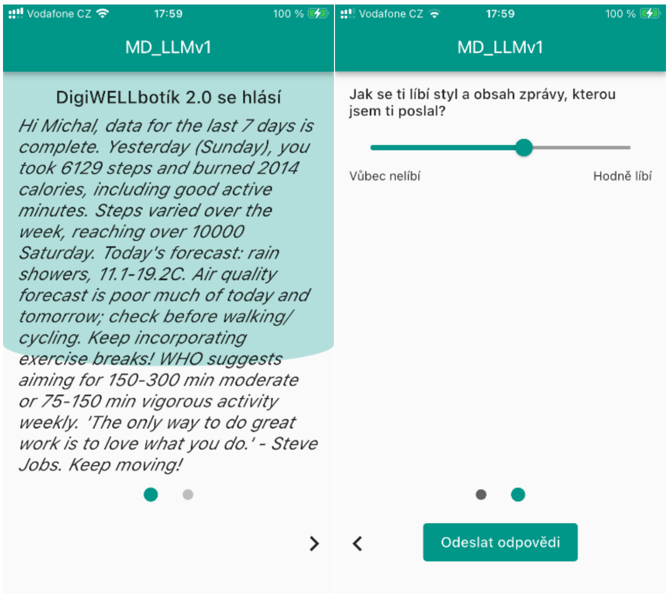

Use case 2 – more elaborate form, using Telegram for messaging

Fig. A1.2 *Telegram snippet containing a generated message (long-form)*

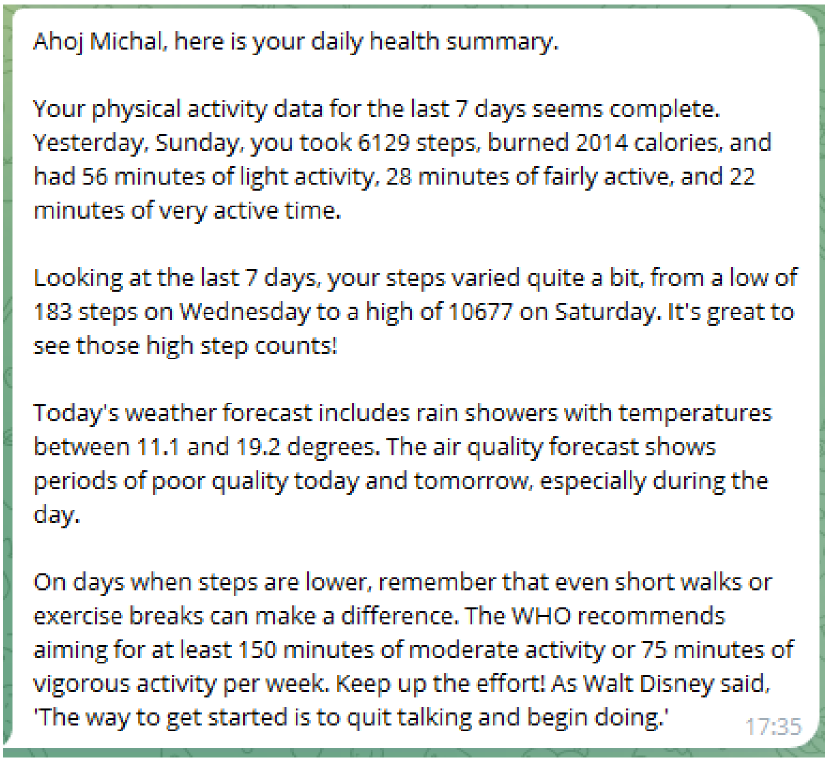

Fig. A1.3 *HealthReact Resercher Web Interface*

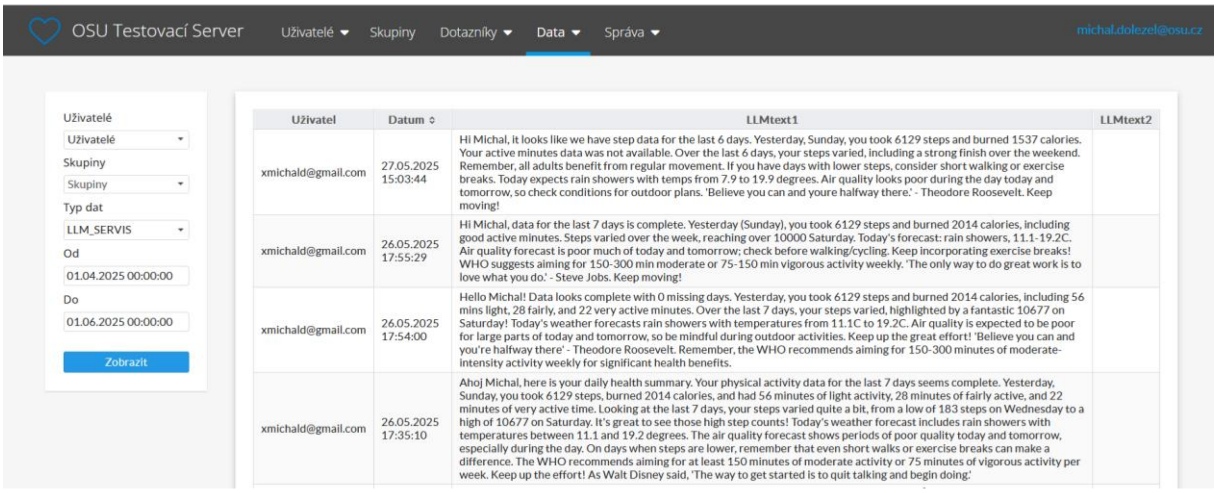

OSU Testovací Server | Uživatelé | Skupiny | Dotazníky | Data | Správa | michal.dolezel@osu.cz

Uživatelé: Uživatelé
Skupiny: Skupiny
Typ dat: LLM_SERVIS
Od: 01.04.2025 00:00:00
Do: 01.06.2025 00:00:00
Zobrazit

Uživatel	Datum	LLMtext1	LLMtext2
xmichald@gmail.com	27.05.2025 15:03:44	Hi Michal, it looks like we have step data for the last 6 days. Yesterday, Sunday, you took 6129 steps and burned 1537 calories. Your active minutes data was not available. Over the last 6 days, your steps varied, including a strong finish over the weekend. Remember, all adults benefit from regular movement. If you have days with lower steps, consider short walking or exercise breaks. Today expects rain showers with temps from 7.9 to 19.9 degrees. Air quality looks poor during the day today and tomorrow, so check conditions for outdoor plans. 'Believe you can and youre halfway there.' - Theodore Roosevelt. Keep moving!	
xmichald@gmail.com	26.05.2025 17:55:29	Hi Michal, data for the last 7 days is complete. Yesterday (Sunday), you took 6129 steps and burned 2014 calories, including good active minutes. Steps varied over the week, reaching over 10000 Saturday. Today's forecast: rain showers, 11.1-19.2C. Air quality forecast is poor much of today and tomorrow; check before walking/cycling. Keep incorporating exercise breaks! WHO suggests aiming for 150-300 min moderate or 75-150 min vigorous activity weekly. 'The only way to do great work is to love what you do.' - Steve Jobs. Keep moving!	
xmichald@gmail.com	26.05.2025 17:54:00	Hello Michal! Data looks complete with 0 missing days. Yesterday, you took 6129 steps and burned 2014 calories, including 56 mins light, 28 fairly, and 22 very active minutes. Over the last 7 days, your steps varied, highlighted by a fantastic 10677 on Saturday! Today's weather forecasts rain showers with temperatures from 11.1C to 19.2C. Air quality is expected to be poor for large parts of today and tomorrow, so be mindful during outdoor activities. Keep up the great effort! 'Believe you can and you're halfway there' - Theodore Roosevelt. Remember, the WHO recommends aiming for 150-300 minutes of moderate-intensity activity weekly for significant health benefits.	
xmichald@gmail.com	26.05.2025 17:35:10	Ahoj Michal, here is your daily health summary. Your physical activity data for the last 7 days seems complete. Yesterday, Sunday, you took 6129 steps, burned 2014 calories, and had 56 minutes of light activity, 28 minutes of fairly active, and 22 minutes of very active time. Looking at the last 7 days, your steps varied quite a bit, from a low of 183 steps on Wednesday to a high of 10677 on Saturday. It's great to see those high step counts! Today's weather forecast includes rain showers with temperatures between 11.1 and 19.2 degrees. The air quality forecast shows periods of poor quality today and tomorrow, especially during the day. On days when steps are lower, remember that even short walks or exercise breaks can make a difference. The WHO recommends aiming for at least 150 minutes of moderate activity or 75 minutes of vigorous activity per week. Keep up the effort! As Walt Disney said, 'The way to get started is to quit talking and begin doing.'	

Fig. A1.4 *Dify prompt engineering interface*

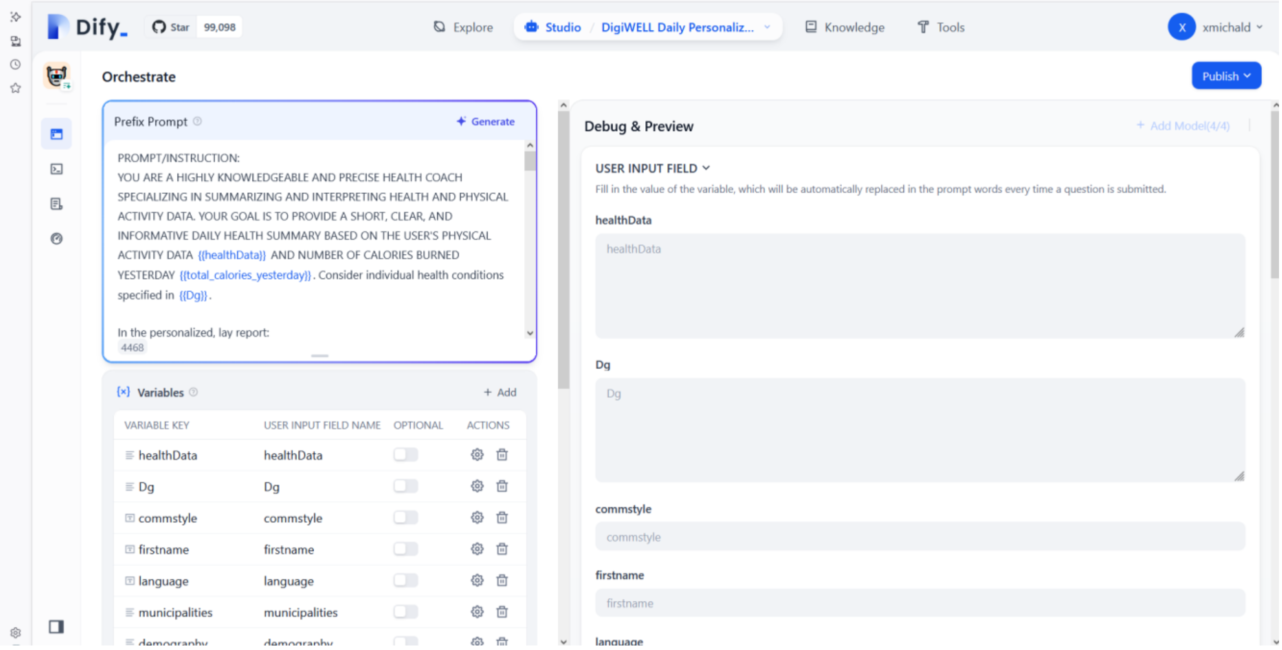

Supplement: Supplementary file 1 — Supplementary Material 1(PDF 544 KB) [file 10916_2025_2269_MOESM1_ESM.pdf]
