# Supplementary material for: Reference and Solution Architecture for GenAI- and GIS-Enhanced Physical Activity Interventions: Towards Implementing the AI4Motion Platform
Source: J Med Syst. 2025 Oct 30;49(1):150. doi: 10.1007/s10916-025-02269-x (PMC12575550; doi:10.1007/s10916-025-02269-x)
Supplement: Supplementary file 2 — Supplementary Material 2(PDF 80.4 KB) [file 10916_2025_2269_MOESM2_ESM.pdf]

On-line appendix 2: GIS in DBCIs

This appendix provides a brief overview of key geographic concepts and GIS functionalities, acknowledging the complexity and interdisciplinary challenges of integrating spatial context into DBCI and LLM technologies.

Central to this integration are frameworks from Time Geography and Health Geography [A1], which offer theoretical grounding for understanding individuals' spatial routines and environmental constraints.

From an operational perspective, integration is enabled through data collection via Location-Aware Technologies (LAT), such as GNSS (Global Navigation Satellite System) modules, embedded in smartphones or wearables [A2]. Combined with GIS, these technologies support the application of methods like Geographic Momentary Assessment (GMA) and Geographically Explicit Ecological Momentary Assessment (GEMA) [A3], which support real-time, spatially specific monitoring and can be integrated into systems that deliver context-aware feedback.

Simultaneously, LBS personalizes content delivery based on the user's current location, drawing on geographic databases such as OpenStreetMap to provide contextual data (e.g., nearby amenities), which enhances personalization and relevance within DBCIs [A4].

Table A1 outlines the key GIS-based concepts and functionalities applied in the DigiWELL LLM-driven DBCI pilot to support context-aware behavior change.

Table A1 *Overview of the key GIS-based concepts and functionalities*

Geographic Feature in DBCI	Description (in overall DBCI context)	Description (in DigiWELL LLM-driven DBCI)	Example of context-aware message
**Geo-fencing and Location-based triggering**	Geofencing and location-based triggering use user location to activate context-aware functions in DBCIs.<br><br>[A5, A6]	Used to deliver context-aware DBCI elements when participants enter, leave, or remain in defined locations for a certain duration.	Still at your workplace? A quick stretch could boost your energy and concentration.
**Geographic Context / Environment**	Incorporates static geographic features (e.g., parks, school zones) and dynamic environmental conditions (e.g., weather, air quality) to tailor timing and	LLM-generated suggestions are tailored to the location type and current environmental conditions (e.g., promoting indoor activities during poor	Rain is expected in your area today. An indoor activity might be the best way to stay on track.

	content of DBCI messages.<br><br>[A7]	weather or low air quality)	
**Point of Interest (POI)**	Integrates known landmarks or venues into DBCIs to tailor suggestions based on local opportunities, such as parks, fitness centers, or culturally significant sites.<br><br>[A4]	LLM-generated messages recommend nearby POIs to promote behavior change, such as walking to a park or visiting a calming spot.	There's a remarkable heritage tree 300 meters from your current location. It could be a peaceful destination for a short walk today.
**Semantic Location Mapping / Semantic Enrichment**	Transforms raw location measurements data into natural language (e.g., 'home', 'gym', 'store'), enabling more relevant and interpretable DBCI logic.<br><br>[A8, A9]	Provides LLMs with semantically enriched location labels, allowing the model to understand where the participant is and generate context-appropriate messages.	Looks like you spent most of yesterday at home. Today could be a good opportunity to get some fresh air around Brno.
**Spatiotemporal Clustering**	Identifies patterns by grouping locations and times that share similar behavioral characteristics, such as recurring physical activity in specific areas during certain times of day.<br><br>[A10]	Supports time-and-location-based suggestions by allowing the LLM to reference habitual patterns in the participant's behavior.	Since you regularly ride public transport, getting off at Antonínská instead of Pionýrská could be an easy and effective way to add a few more active minutes to your day.

Appendix References

A1. Barber BV, Kephart G, Martin-Misener R, et al (2024) Integrating health geography and behavioral economic principles to strengthen context-specific behavior change interventions. Transl Behav Med 14:257–272. https://doi.org/10.1093/tbm/ibad065

A2. Miller HJ (2010) The Data Avalanche Is Here. Shouldn't We Be Digging? J Reg Sci 50:181–201. https://doi.org/10.1111/j.1467-9787.2009.00641.x

A3. Zhang Y, Li D, Li X, et al (2024) The integration of geographic methods and ecological momentary assessment in public health research: A systematic review of methods and applications. Soc Sci Med 354:117075. https://doi.org/10.1016/j.socscimed.2024.117075

A4. Huang H, Gartner G (2012) Using Context-Aware Collaborative Filtering for POI Recommendations in Mobile Guides. In: Gartner G, Ortag F (eds) Advances in Location-Based Services. Lecture Notes in Geoinformation and Cartography. Berlin, Heidelberg, pp 131–147

A5. Tobin K, Heidari O, Volpi C, et al (2023) Use of geofencing interventions in population health research: a scoping review. BMJ Open 13:e069374. https://doi.org/10.1136/bmjopen-2022-069374

A6. Törnros T, Dorn H, Reichert M, et al (2016) A comparison of temporal and location-based sampling strategies for global positioning system-triggered electronic diaries. Geospat Health 11:917–1506. https://doi.org/10.4081/gh.2016.473

A7. Kyung N, Chan J, Lim S, Lee B (2024) Contextual Targeting in mHealth Apps: Harnessing Weather Information and Message Framing to Increase Physical Activity. Information Systems Research 35:1034–1051. https://doi.org/10.1287/isre.2020.0119

A8. Ibrahim A, Zhang H, Clinch S, Harper S (2021) From GPS to semantic data: how and why—a framework for enriching smartphone trajectories. Computing 103:2763–2787. https://doi.org/10.1007/s00607-021-00993-z

A9. Muroň M, Dařena F, Procházka D, Kern R (2025) Automatically generated place descriptions for accurate location identification: a hybrid approach with rule-based methods and LLM. Spat Cogn Comput 1–43. https://doi.org/10.1080/13875868.2025.2449859

A10. Boytsov A, Zaslavsky A, Abdallah Z (2012) Where Have You Been? Using Location Clustering and Context Awareness to Understand Places of Interest. In: Andreev S, Balandin S, Koucheryavy Y (eds) Internet of Things, Smart Spaces, and Next Generation Networking. ruSMART NEW2AN 2012 2012. Lecture Notes in Computer Science. Springer, Berlin, Heidelberg, pp 51–62
